# Effects of valproic acid on the cell cycle and apoptosis through acetylation of histone and tubulin in a scirrhous gastric cancer cell line

**DOI:** 10.1186/1756-9966-29-149

**Published:** 2010-11-17

**Authors:** Yasumichi Yagi, Sachio Fushida, Shinichi Harada, Jun Kinoshita, Isamu Makino, Katsunobu Oyama, Hidehiro Tajima, Hideto Fujita, Hiroyuki Takamura, Itasu Ninomiya, Takashi Fujimura, Tetsuo Ohta, Masakazu Yashiro, Kosei Hirakawa

**Affiliations:** 1 Department of Gastroenterologic Surgery, Division of Cancer Medicine, Graduate School of Medical Science, Kanazawa University, Ishikawa 920-8641, Japan; 2 Center for Biomedical Research and Education, School of Medicine; Kanazawa University, Ishikawa 920-8641, Japan; 3 Department of Surgical Oncology, Graduate School of Medicine, Osaka City University, Osaka 545-8585, Japan

## Abstract

**Background:**

Management of peritoneal dissemination is the most critical problem in gastric cancer. This study was performed to investigate the inhibitory effects of valproic acid (VPA) on a highly peritoneal-seeding cell line of human scirrhous gastric cancer, OCUM-2MD3, and to explore the mechanism and the potential of VPA.

**Methods:**

The effects of VPA on the growth of OCUM-2MD3 cells were assessed by MTT assay. In addition, paclitaxel (PTX) was combined with VPA to evaluate their synergistic effects. HDAC1 and HDAC2 expression were evaluated by western blotting in OCUM-2MD3 cells and other gastric cancer cell lines (TMK-1, MKN-28). The acetylation status of histone H3 and α-tubulin after exposure to VPA were analyzed by western blotting. The activities of cell cycle regulatory proteins and apoptosis-modulating proteins were also examined by western blotting. The effects of VPA *in vivo *were evaluated in a xenograft model, and apoptotic activity was assessed by TUNEL assay.

**Results:**

OCUM-2MD3 cells showed high levels of HDAC1 and HDAC2 expression compared with TMK-1 and MKN-28. The concentration of VPA required for significant inhibition of cell viability (*P *< 0.05) was 5 mM at 24 h and 0.5 mM at 48 h and 72 h. The inhibition of VPA with PTX showed dose-dependent and combinatorial effects. VPA increased acetyl-histone H3, acetyl-α-tubulin, and p21WAF1 levels accompanied by upregulation of p27, caspase 3, and caspase 9, and downregulation of bcl-2, cyclin D1, and survivin. In the xenograft model experiment, the mean tumor volume of the VPA-treated group was significantly reduced by 36.4%, compared with that of the control group at 4 weeks after treatment (*P *< 0.01). The apoptotic index was significantly higher in the VPA-treated group (42.3% ± 3.5%) than in the control group (7.7% ± 2.5%) (*P *< 0.001).

**Conclusions:**

VPA induced dynamic modulation of histone H3 and α-tubulin acetylation in relation with the anticancer effect and the enhancement of PTX in the OCUM-2MD3 cell line. Therefore, VPA in combination with PTX is expected to be a promising therapy for peritoneal dissemination of scirrhous gastric cancer.

## Background

Gastric cancer remains one of the leading causes of cancer death in the world [1]. Particularly, the prognosis of scirrhous gastric cancer is poorer than those of other types of gastric cancer [2,3]. In gastric cancer, the most critical factor responsible for poor prognosis is peritoneal dissemination. Consequently, the management of peritoneal dissemination is an urgent problem in gastric cancer patients.

The recent development of anticancer drugs and intraperitoneal chemotherapy improved the clinical outcomes in gastric cancer patients with peritoneal dissemination [4,5]. Moreover, molecular targeted therapy has attracted a great deal of attention as a new class of anticancer agents. Clinical studies indicated that combining molecular targeted agent with conventional chemotherapy enhances the inhibition of tumor growth and metastasis in gastric cancer patients [6,7]. Chemosensitivity is influenced by changes in expression of various genes, including those known to be associated with the cell cycle and apoptosis [8]. There is increasing evidence that epigenetic alterations, such as histone acetylation and promoter methylation, play important roles in regulation of gene expression associated with the cell cycle and apoptosis [9]. Chromatin remodeling is physiologically regulated by two enzymes, histone acetyltransferase (HAT) and histone deacetylase (HDAC). The ratio of these two enzymes regulates the amount of histone acetylation and controls posttranslational modification of histones and gene transcription. Acetylation of lysine residues of the histones weakens their binding to DNA and induces a change in DNA conformation essential for binding of transcription factors to the promoter regions of target genes [10,11]. HDACs are subdivided into three classes [12,13]. Class I HDACs are composed of HDAC 1 - 3 and 8. Class II HDACs are composed of HDAC 4 - 7 and 9 - 11. Aberrant levels of HDAC activity have been found in a variety of human malignancies and result in repression of tumor-suppressor genes and promotion of tumorigenesis [14]. HDAC inhibitors represent a structurally diverse group of compounds that inhibit the deacetylation of histones, permitting the chromatin scaffolding to assume a more relaxed, open conformation, which generally promotes gene transcription. Recently, HDAC inhibitors have been shown to have antiproliferative activity through cell cycle arrest, differentiation, and apoptosis in various cancer cell types [15], including gastric cancer cell lines [16,17]. Especially, the combination of HDAC inhibitor with conventional chemotherapy is expected to have a synergistic effect, because the mechanism of action is different from those of conventional chemotherapeutic regimens.

Valproic acid (VPA), which has long been used clinically for treatment of epilepsy and bipolar disorder without significant toxicity, causes hyperacetylation of the N-terminal tails of histones H3 and H4 *in vitro *and *in vivo *and inhibits HDAC activity, probably by binding to the catalytic center and thereby blocking substrate access [18,19]. VPA inhibits both class I and II HDACs, with high potency for class I HDACs [20]. Earlier studies indicated that p21WAF1, one of the target genes induced by VPA, affects differentiation and decreases tumor cell growth [21,22]. Another report focused on the apoptotic activity of VPA [23]. However, the detailed mechanism of apoptosis by VPA has not been elucidated. On the other hand, recent evidence suggests that HDAC inhibitors also enhance the acetylation of non-histone proteins, such as p53, c-Jun, and α-tubulin [24-26]. It is possible that VPA increases acetylation of non-histone proteins in relation with apoptosis. However, no reports have focused on the therapeutic potential of VPA in gastric cancer. The present study was performed to investigate the anticancer mechanism of action of VPA by analyzing the expression of cell cycle regulatory proteins and apoptosis-modulating proteins in a scirrhous gastric cancer cell line. In addition to acetylation of histones, the possibility that acetylation of the non-histone protein α-tubulin contributes to inhibition of tumor growth was also examined.

Paclitaxel (PTX) is an anticancer agent, which stabilizes polymerized microtubules and enhances microtubule assembly, and thus arrests the cell cycle in G0/G1 and G2/M phases, leading to cell death [27], and has been used for peritoneal dissemination of ovarian and gastric cancer [4,28]. As tubulin is a target molecule of PTX, combination of VPA with PTX has the potential to show synergistic effects. In the present study, we also evaluated the synergistic effects of PTX with VPA on a scirrhous gastric cancer cell line. The mechanisms of these anticancer effects of VPA, which are different from conventional chemotherapy, may provide a new strategy to improve the clinical outcome of gastric cancer patients.

## Methods

### Materials

VPA was purchased from Sigma-Aldrich Co. (Japan). PTX was kindly provided by Bristol-Myers Squibb Company (Japan).

### Cell lines and cell culture

OCUM-2MD3, a highly peritoneal-seeding cell line derived from human scirrhous gastric cancer, was kindly provided by the Department of Surgical Oncology of Osaka City University of Medicine. In this study, we used mainly OCUM-2MD3 to investigate the efficacy of VPA on this peritoneal-seeding cell line. Other human gastric cancer cell lines (MKN28, moderately differentiated adenocarcinoma; TMK-1, poorly differentiated adenocarcinoma) were obtained from the American Type Culture Collection (Rockville, MD). These were seeded in 75-cm^2 ^dishes (Becton Dickinson, Japan) and cultured in 10 mL of medium at 37°C in a humidified atmosphere of 5% CO_2 _in air. OCUM-2MD3 cells were grown in DMEM (Invitrogen, Japan) supplemented with 10% heat-inactivated fetal bovine serum (Nichirei Bioscience Inc., Japan), 100 IU/mL penicillin, 100 mg/mL streptomycin (Invitrogen), 2 mM glutamine (Nissui Pharmaceutical Co., Ltd., Japan), and 0.5 mM sodium pyruvate. The culture medium for MKN28 and TMK-1 cells was RPMI (Nissui) with the same additives as above. Cells were grown to confluence and harvested by trypsinization with 0.25 mg/mL trypsin/EDTA (Invitrogen) and suspended in culture medium before use.

### Cell growth assay

The viability of OCUM-2MD3 cells treated with VPA was determined by standard 3-(4, 5-Dimethylthiazol-2-yl)-2, 5-diphenyltetrazolium bromide (MTT) assay. OCUM-2MD3 cells were seeded at 5 × 10^3 ^per well in 96-well plates and incubated overnight at 37°C. After incubation, the supernatant was discarded and replaced with fresh serum-free culture medium. VPA was dissolved in phosphate buffered saline (PBS) and added to the cell culture medium at various concentrations (0 - 10 mM). At 24, 48, and 72 h after exposure to VPA, the supernatant was discarded and MTT solution was added to each well (500 μg/mL final concentration) and incubated at 37°C for 3 h. Then, the supernatant was removed, and 150 μL of DMSO was added. The absorbance of the solution was read at a wavelength of 540 nm using a microplate reader (BIO-RAD550; BIO-RAD, Japan). The percentage inhibition was determined by comparing the cell density of the drug-treated cells with that of untreated controls. All experiments were repeated at least three times. In addition, the effects of VPA combined with PTX were evaluated at various concentrations.

### Animals and xenograft model treated with VPA

BALB/c *nu/nu *mice (female, 4 - 6 weeks old; Charles River Laboratories, Japan, Inc) were used for xenograft models. They were housed under specific pathogen-free conditions and fed standard chow pellets and water *ad libitum*. Experiments were performed according to the Standard Guidelines for Animal Experiments of Kanazawa University. The effects of VPA on the xenograft model were examined as follows: OCUM-2MD3 cells (2 × 10^6 ^cells) were inoculated s.c. into the dorsal side of mice. The mice were divided into two groups: a control group (PBS i.p., *n *= 6) and a VPA-treated group (10 mg/mouse i.p. for 5 days per week, *n *= 6). The treatment was started on day 7 after xenografting and discontinued after 5 weeks. Tumors were measured weekly with Vernier calipers. Tumor volume was calculated using the following formula: *volume *= *length *× *width *× *height *× *0.5236*. At the end of the experiment, tumor specimens were collected for immunohistochemical examination and TUNEL assay.

### Western blotting

The effects of VPA on acetylation of histone H3 and α-tubulin, cell cycle regulatory and apoptosis-related proteins, were analyzed in cell lysates by western blotting. OCUM-2MD3 cells were seeded at a density of 1 × 10^6 ^cells per 75-cm^2 ^dish and cultured in 10 mL of medium overnight. Lysates were obtained from the cells harvested at 0, 0.5, 1, 3, 6, 12, 24, and 48 h after incubation with 1 mM VPA, which corresponded approximately to the level obtained by administrating a clinical dose of VPA. Whole-cell lysates were prepared in denaturing SDS sample buffer and subjected to SDS-PAGE (ATTO Co. Ltd., Japan). As primary antibodies, a rabbit polyclonal HDAC1 antibody (1:5000) (Santa Cruz Biotechnology Inc., Santa Cruz, CA), rabbit polyclonal HDAC2 antibody (1:5000) (Santa Cruz), rabbit polyclonal acetyl-histone H3 (Lys 9) antibody (1:5000) (Cell Signaling, Beverly, MA), mouse monoclonal acetyl α-tubulin antibody (1:5000) (Sigma), and mouse monoclonal β-actin antibody (1:5000) (Sigma) were used. As antibodies against apoptosis-related proteins, a rabbit polyclonal cleaved caspase 3 (Asp175) antibody (1:5000) (Cell Signaling), mouse monoclonal caspase 9 antibody (1:5000) (Santa Cruz), mouse monoclonal bcl-2 antibody (1:5000) (Santa Cruz), mouse monoclonal survivin 6E4 antibody (1:5000) (Cell Signaling), and mouse monoclonal p53 antibody (1:5000) (Sigma) were used. As antibodies against cell cycle regulatory proteins, a mouse monoclonal p21WAF1 (1:5000) (Pharmingen, San Diego, CA), mouse monoclonal p27 antibody (Santa Cruz), and mouse monoclonal cyclin D1 (1:5000) (Sigma) were used. The immunoblots were visualized using an ECL Plus kit (GE Healthcare UK Ltd., Japan). The antibody-antigen complex was detected using an ECL Western-Blotting detection kit (GE Healthcare) and the Light-Capture system (ATTO) and then quantified using the CS analyzer program (ATTO).

### Immunohistochemical examination and TUNEL assay

Tumor specimens obtained from xenograft models were fixed in 10% neutral buffered formalin and embedded in paraffin. The sections were stained with H&E and immunostained with a mouse monoclonal p21WAF1 (1:200) (Pharmingen) and a rabbit polyclonal cleaved caspase 3 antibody (1:200) (Cell Signaling) at 4°C overnight. The sections were reacted with EnVision reagent (Dako Co., Japan) for visualization. The degree of apoptosis was evaluated using the TdT-mediated dUTP nick-end labeling (TUNEL) method (Apoptosis *in situ *Detection Kit; Wako, Osaka, Japan). For quantitative analysis, the cells that were TUNEL-positive and also fulfilled the morphological criteria of apoptosis were counted under ×400 magnification in 10 randomly chosen fields representing at least 1000 nuclei. The results were expressed as the mean percentage of apoptosis cells. These results were used as the apoptotic index (*n *= 6 in each group).

### Statistical analysis

Values are expressed as means ± standard deviation (SD). Comparisons among the data sets were made by Student's *t *test using the computer software package SPSS10.0 (SPSS, Japan, Inc). In all analyses, *P *< 0.05 was taken to indicate statistical significance.

## Results

### Expression of HDAC1 and HDAC2 in OCUM-2MD3 cells

**Figure 1:**
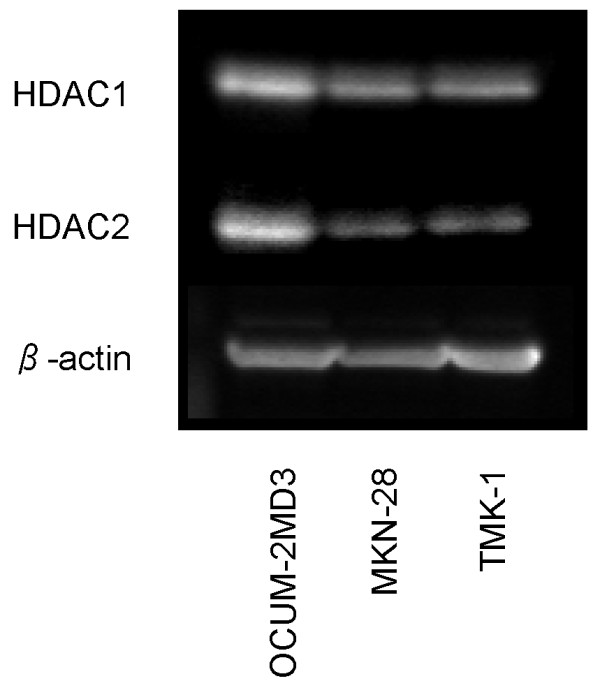
**Expression of HDAC1 and HDAC2 in gastric cancer cell lines examined by western blotting**. OCUM-2MD3 showed high levels of HDAC1and 2 compared with other cell lines.

**Figure 2:**
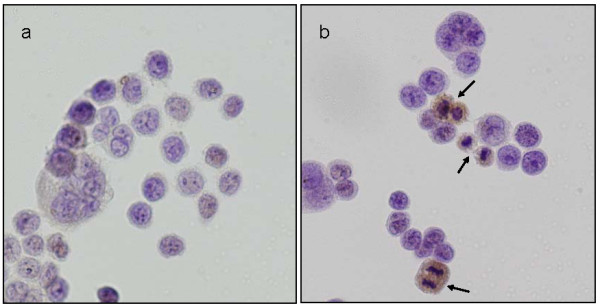
**Immunostaining of HDAC1 and HDAC2 in OCUM-2MD3 cells**. Both HDAC1 and HDAC2 were expressed mainly in nuclei of tumor cells. Expression of HDAC2 was observed characteristically in tumor cells in the mitotic period (arrows). (a) HDAC1, (b) HDAC2. Original magnification ×400.

On western blotting analysis, OCUM-2MD3 cells showed high levels of HDAC1 and HDAC2 compared with the other human gastric cancer cell lines (Figure 1). Immunohistologically, both HDAC1 and HDAC2 were expressed mainly in nuclei. The HDAC2 expression was characteristically observed in the cells in mitotic phase (Figure 2).

### Effects of VPA on the growth of OCUM-2MD3 cells *in vitro*

**Figure 3:**
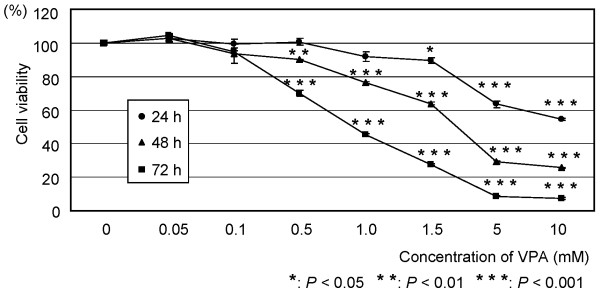
**Effects of VPA on the growth of OCUM-2MD3 cells *in vitro***. Cell viability was assessed by MTT assay. OCUM-2MD3 cells were treated with the indicated doses of VPA (0 - 10 mM) in serum-free medium.

**Figure 4:**
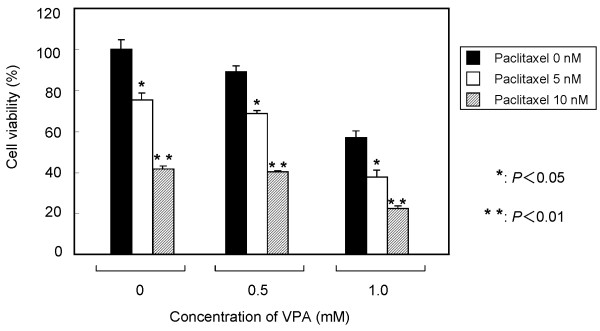
**Combinatorial effects of VPA with PTX *in vitro***. The results are means ± SD of three different experiments.

As shown in Figure 3, the inhibition of VPA in OCUM-2MD3 cells was dependent on the dose and incubation time. The concentration of VPA required for significant inhibition of cell viability (*P *< 0.05) was 5 mM at 24 h, and 0.5 mM at 48 h and 72 h. Degenerated cancer cells were observed at high concentrations (> 5 mM at 48 and 72 h) of VPA (data not shown). According to these results, we examined the western blotting by 1 mM VPA, which showed the evident decrease of OCUM-2MD3 cells. VPA in combination with PTX showed dose-dependent combinatorial effects (Figure 4).

### Effects of VPA on acetyl-histone H3 level, cell cycle regulatory protein

**Figure 5:**
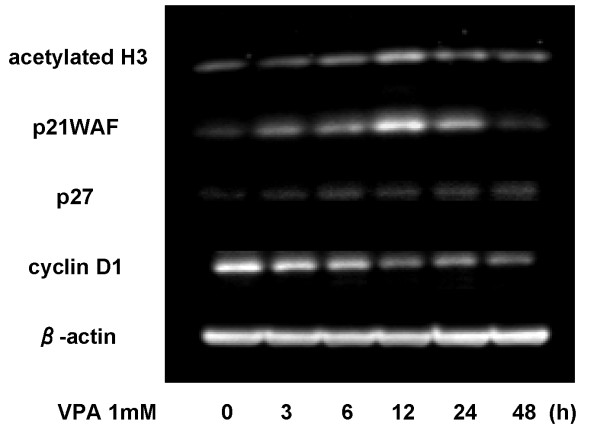
**Time courses of changes in protein levels, including acetyl-histone H3, cell cycle regulatory proteins (p21WAF1, p27 and cyclin D1)**. OCUM-2MD3 cells were treated with 1 mM VPA, and cell lysates were harvested up to 48 h. Western blotting was performed using a series of primary antibodies.

The acetylation status of histone H3 in OCUM-2MD3 cells was determined during 48 h of incubation with 1 mM VPA, using an antibody that specifically recognizes hyperacetylated forms of histone H3. As shown in Figure 5, VPA markedly increased acetyl-histone H3 expression with maximal induction at 12 h of incubation with VPA. In addition, the maximal increase of p21WAF1 was detected concomitant with activation of acetyl-histone H3. The level of p27 showed a gradual increase for up to 48 h. In contrast, VPA showed a gradual decrease in cyclin D1 level.

### Effects of VPA on the induction of apoptosis

**Figure 6:**
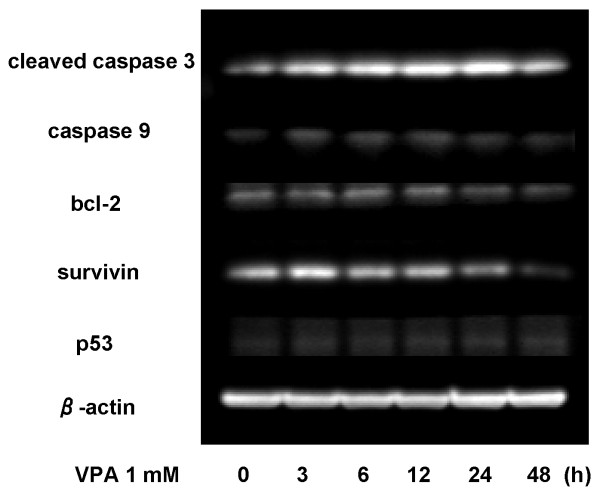
**Time courses of changes in apoptosis-related proteins**. Cleaved caspase 3, caspase 9, survivin, bcl-2 and p53 were examined by western blotting with a series of primary antibodies. Lysates were obtained from OCUM-2MD3 cells with exposure to 1 mM VPA up within 48 h incubation.

We analyzed the effects of VPA on apoptotic regulatory proteins by western blotting (Figure 6). The levels of cleaved caspase 3 and caspase 9 showed mild increases up to 24 h, suggesting that the apoptosome pathway was activated by this VPA treatment. Conversely, the levels of bcl-2 and survivin gradually decreased. VPA reduced bcl-2 level by 30% and survivin level by 70%, suggesting that the antiapoptotic activity was suppressed by this HDAC inhibitor.

### Acetylation of tubulin after exposure to VPA

**Figure 7:**
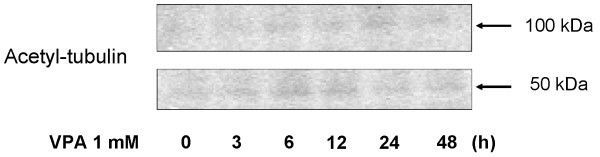
**Acetylation status of α-tubulin assessed by western blotting**. Acetyl-α-tubulin level was increased after exposure to 1 mM VPA. 50 kDa: monomer; 100 kDa: dimer.

Figure 7 shows the status of tubulin acetylation determined by western blotting. Increased acetyl-α-tubulin was detected by 6 h and the maximal induction was evident by 12 h. Such rapid tubulin acetylation occurred in parallel with increases in acetyl-histone H3 and p21WAF1.

### Effects of VPA on xenograft model *in vivo*

**Figure 8:**
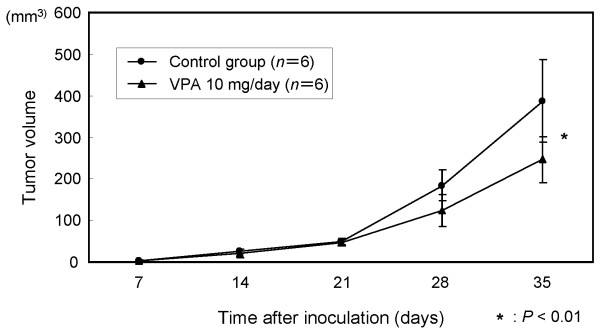
***In vivo *effects of VPA on the growth of tumor xenografts**. The results are means ± SD of three different experiments.

**Figure 9:**
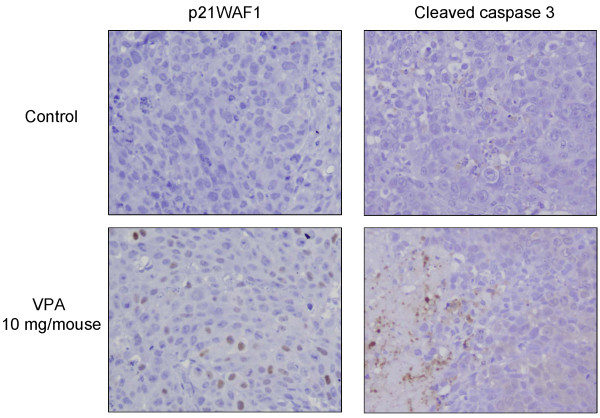
**Effects of VPA on the expression of p21WAF1 and cleaved caspase 3 in xenograft model**. Immunohistochemical examination showed that p21WAF1-positive cells (nuclear staining) were increased compared with the control group. Cleaved-caspase 3-positive cells were observed as apoptotic cells characterized by cell shrinkage and nuclear fragmentation in the VPA-treated group. Original magnification ×400.

**Figure 10:**
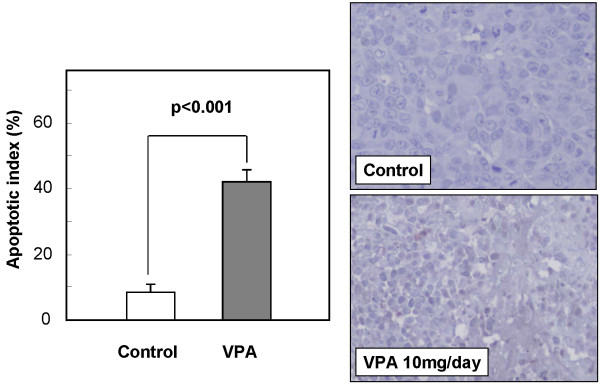
**Effects of VPA on apoptosis in the xenograft model**. Shrunken tumor cells showed positive reactivity in TUNEL assay. Apoptotic index of the VPA-treated group was significantly higher than that of the control group. Original magnification ×400.

The time courses of changes in xenografted tumor volume are shown in Figure 8. The mean tumor volume of the VPA-treated group (246.3 ± 56.0 mm^3^) was significantly reduced by 36.4%, compared with that of the control group (387.5 ± 99.6 mm^3^) at 4 weeks after treatment (*P *< 0.01). As shown in Figure 9, immunohistochemical examination of the xenografted tumor revealed upregulation of p21WAF1 in the VPA-treated group. Moreover, degenerated cells with VPA treatment showed reactivity for cleaved caspase 3, indicating caspase 3 activation. TUNEL assay showed that the apoptotic index was significantly higher in the VPA-treated group (42.3% ± 3.5%) than in the control group (7.7% ± 2.5%) as shown in Figure 10 (*P *< 0.001).

## Discussion

The results of the present study showed that VPA alone has an antiproliferative effect on a scirrhous gastric cancer cell line (OCUM-2MD3) *in vitro *and *in vivo*. VPA increased the acetylation of histone H3, resulting in a significant reduction of tumor growth through induction of both p21WAF1 and apoptosis. Furthermore, we also demonstrated that VPA induces α-tubulin acetylation, thus stabilizing tubulin, suggesting that VPA in combination with PTX will have a synergistic effect.

Previous studies showed that the HDAC inhibitor trichostatin A has an antiproliferative effect through cell cycle regulation and apoptosis [16], and increases chemosensitivity of gastric cancer cell lines to anticancer drugs, including 5-fluorouracil, PTX, and irinotecan [17]. In the present study, the acetylation of histone H3 was observed with upregulation of p21WAF1 expression, supporting the suggestion that VPA induces differentiation of cancer cells as reported previously [29]. In addition, VPA induced alterations in the expression of other cell cycle-related proteins, such as p27 and cyclin D1. As p21WAF1 and p27 are cyclin-dependent kinase inhibitors that bind to cyclin-dependent kinase complexes and decrease kinase activity, they may act as key regulators of G_0_/G_1 _accumulation [30]. Most previous studies indicated that HDAC inhibitors upregulate the transcription of p53 [31,32]. However, Sami *et al*. reported the efficacy of VPA with no effect on p53 expression [18]. In the present study, we also demonstrated that VPA has an anticancer effect through a p53-independent pathway. With regard to apoptosis, the activation of caspase-3 and caspase-9 and the downregulation of bcl-2 and survivin were observed with the apoptotic activity induced by VPA in the present study. Taken together, the total effects on the cell cycle and apoptosis were considered to result in the anticancer activity of VPA.

Peritoneal dissemination of scirrhous gastric cancer is characterized by rapid infiltration and proliferation of cancer cells with abundant fibrosis in the stroma [33]. From the viewpoint of molecular biology, transforming growth factor-β (TGF-β) is considered a key factor, which contributes to the invasiveness and morphological changes in peritoneal dissemination of diffuse-type gastric cancer [34]. Clinically, the expression of TGF-β is correlated to the malignant potential of scirrhous gastric cancer [35]. It has also been reported that TGF-β produced by stromal fibroblasts or gastric cancer cells stimulates both the invasion and adhesion of scirrhous gastric cancer cells to the peritoneum, resulting in an increase in the potential for peritoneal dissemination [36,37]. On the other hand, TGF-β is considered a major factor that triggers epithelial-mesenchymal transition (EMT), which promotes invasion and metastasis with acquiring fibroblastoid features and morphological changes [38-40]. Accordingly, TGF-β-induced EMT could be a target for regulation of aggressiveness in gastric cancer. These changes induced by TGF-β may work better for formation of peritoneal dissemination. On the other hand, HDAC4 plays an essential role in epigenetic regulation of myofibroblastic differentiation by TGF-β induction [41]. HDAC4 could be a target for interstitial fibrosis involved in peritoneal dissemination. In addition, VPA can also inhibit an activity of HDAC4 which is one of class II HDACs [29]. Therefore, VPA has the potential to reduce fibrosis by inhibition of HDAC4. However, further investigations are needed to confirm the effectiveness of VPA on fibrosis.

We found that VPA increases acetylation of α-tubulin as well as histone H3. Interestingly, tubulin acetylation has a direct relation with HDAC6 inhibition induced by the action of VPA [42,43]. HDAC inhibitors also play a role as microtubule-associated deacetylases and cause acetylation of lysine40 of α-tubulin [44,45]. Acetylation of tubulin may contribute to the inhibition of tumor cell growth in addition to the known effects caused by histone acetylation. On the other hand, the mechanism of tubulin acetylation by HDAC inhibitors could have a favorable effect in combination with PTX [26,46], which is a key drug in the treatment of gastric cancer. As PTX is a taxane-based drug that interferes with mitosis and cell replication by binding to a subunit of tubulins, PTX has the potential to reduce fibrosis by inhibition of TGF-β/Smad signaling [47-50]. It is noteworthy that the inhibition of tumor cell proliferation can be achieved by much higher dosages of PTX. In contrast, the inhibition of TGF-β/Smad signaling can be attained with very low doses of PTX [47]. Therefore, we suggest that VPA enhances the anticancer action in combination with PTX. However, further clinical studies are required to determine the clinical applicability of the combination treatment.

VPA is a safe drug with excellent bioavailability based on long-term clinical experience in the treatment of epilepsy. Recent clinical trials for various malignancies have shown that the serum concentration of VPA, achieved during therapy of epilepsy with a daily dose, acts as a potent inhibitor of HDACs required for histone acetylation [51,52]. Biomonitoring of peripheral blood lymphocytes demonstrated the induction of histone hyperacetylation in the majority of patients and downregulation of HDAC2 [51]. In addition to the antitumor effect, VPA plays a variety roles as a mood-stabilizer and analgesic adjuvant for patients in advanced stages of malignancies [53,54]. However, continuous oral treatment with VPA at high doses is not feasible for patients with advanced stages of cancer due to gastrointestinal disturbance [55,56]. Further development of VPA as an HDAC inhibitor in patients with gastric cancer requires careful consideration of the treatment schedule and synergism with conventional chemotherapy. Class I HDAC is overexpressed in gastric cancer patients [57,58]. Both HDAC1 and HDAC2 play important roles in the aggressiveness and carcinogenesis of gastric cancer [59,60]. High levels of expression of class I HDACs, especially HDAC2, are clinically associated with nodal spread and prognosis of gastric cancer patients [61]. These relationships suggest that the level of class I HDAC is a reliable maker of prognosis and a specific target for VPA treatment. Moreover, the effect of VPA, which is a class I- and class II- specific HDAC inhibitor, may depend on the expression patterns of HDACs in tumor cells. The availability of VPA in patients with gastric cancer may depend on patient selection based on biological parameters, such as HDAC2 overexpression. Under pathological conditions of peritoneal dissemination characterized by fibrosis, HDAC4 also may be a target of VPA.

## Conclusion

Our data suggested that VPA induces dynamic modulation of histone and tubulin acetylation, in relation to the anticancer effect and the enhancement of PTX. The multifunctional effect of VPA provides insight into the design of suitable drug combination therapies, including microtubule targeting drugs. Therefore, the combination of VPA and PTX is expected to be a promising regimen in cases of peritoneal dissemination of gastric cancer.

## Competing interests

The authors declare that they have no competing interests.

## Authors' contributions

YY carried out most of experiments, participated in the design of the study, performed the statistical analysis and drafted the manuscript. SF, SH and JK participated in the design of the study and helped to draft the manuscript. IM, KO, HT and HF assisted the experiments. HT, IN, TF, TO, MY and KH participated in its design and coordination. All authors read and approved the final manuscript.
